# Exploring barriers and drivers to a modified WHO safe childbirth checklist implementation in three West African countries: a qualitative study using the updated consolidated framework for implementation research

**DOI:** 10.3389/frhs.2025.1593083

**Published:** 2025-06-19

**Authors:** Kadidiatou Raïssa Kourouma, Wambi Maurice Evariste Yaméogo, Daouda Doukouré, Alpha Oumar Sall, Marie Laurette Agbré Yacé, Tiéba Millogo, Mamadou Diouldé Baldé, Issaka Tiembré, Alexandre Delamou, Séni Kouanda

**Affiliations:** ^1^ National Institute of Public Health (INSP), Abidjan, Côte d'Ivoire; ^2^ Centre for Reproductive Health Research of Côte D’Ivoire (CRESARCI), Abidjan, Côte D'Ivoire; ^3^ African Institute of Public Health, Ouagadougou, Burkina Faso; ^4^ Research Institute for Health Sciences (IRSS), Ouagadougou, Burkina Faso; ^5^ Centre for Reproductive Health Research of Guinea (CERREGUI), Conakry, Guinea; ^6^ Department of Biomedical and Public Health, Faculty of Medicine, University Félix Houphouët-Boigny, Abidjan, Côte D'Ivoire; ^7^ Department of Public Health, African Center of Excellence for the Prevention and Control of Communicable Diseases (CEA-PCMT), Gamal Abdel Nasser University, Conakry, Guinea

**Keywords:** quality of care, safe childbirth checklist, implementation science, barriers, drivers, West Africa

## Abstract

**Introduction:**

Sub-Saharan Africa faces the highest maternal and newborn mortality and morbidity rates globally. The World Health Organization Safe Childbirth Checklist (WHO SCC) was developed to address this issue by promoting evidence-based practices during childbirth. This study explored the barriers and drivers to implementing a modified WHO SCC (mSCC) in Burkina Faso, Cote d'Ivoire, and Guinea.

**Methods:**

A qualitative multiple case study design was conducted from May to June 2023, involving individual interviews with diverse stakeholders (*n* = 110) across four regional hospitals in each country. The mSCC was implemented in these hospitals along with training and coaching. Data was analyzed using thematic analysis, guided by the updated CFIR framework. Nvivo 14 was used for coding.

**Results:**

The study identified 17 drivers and 7 barriers. Key drivers included the mSCC's clarity, simplicity and alignment with national guidelines, training, coaching, and stakeholders’ engagement. in these 3 countries. Barriers were mainly related to resource constraints (medicines, supplies, staffing, and space), increased workload, and lack of incentives. Specific barriers for Burkina Faso and Cote d’Ivoire were the lack of incentives and the positioning of the Kakemono in confined space only in Cote d'Ivoire. Despite these challenges, the mSCC was generally well-received, with strong support from leadership and implementation facilitators contributing to its integration into routine care.

**Conclusion:**

This study highlighted the importance of addressing resource limitations, optimizing workload, and providing incentives to ensure successful mSCC implementation. Findings underscored the need for context-specific strategies and strong leadership support when introducing similar interventions in resource-constrained settings.

## 1 Introduction

Women and newborn of sub-Saharan Africa countries face the highest risk of mortality and morbidity of any region in the world, around the time of delivery (1, 2). The WHO Safe Childbirth Checklist (WHO SCC) was developed especially for developing countries to ensure the delivery of essential maternal and perinatal care practices around the time of childbirth. The WHO SCC is an organized list of evidence-based essential birth practices, which targets the major causes of maternal deaths, intrapartum-related stillbirths and neonatal deaths that occur in healthcare facilities. Each WHO SCC item is a critical action that, if missed, can lead to severe harm for the mother, the newborn, or both (3).

Some Sub Saharan African countries participated in the WHO process to develop and test the tool for adoption by frontline healthcare providers (4–6). The tool was tested and approved for use in delivery rooms in selected countries in Africa (Rwanda, Namibia), Asia (Sri Lanka, India, Bangladesh) and Latin America (Brazil) (4–14). Therefore, a multi-country study to assess the feasibility, acceptability and effectiveness of the WHO SCC was conducted in Cote d'Ivoire, Burkina Faso and Guinea (15–17).

Although many studies have explored the barriers and enablers of the WHO SCC implementation (5, 7), west African countries may encounter unique challenges and opportunities due to their local contexts, characterized by specific maternal socio-economic factor, health indicators, maternal health challenges. access to care disparities, varying levels of health literacy within the population, and communication styles between healthcare providers and patients. Additionally, there is a scarcity of research in West Africa, with no prior studies focusing specifically on the barriers and enablers of the WHO SCC implementation (5). Therefore, we undertook a qualitative study to explore the barriers and drivers of the mSCC implementation in Burkina Faso, Cote d’Ivoire and Guinea.

## 2 Description of the intervention

Before implementing the WHO SCC, we conducted the feasibility and acceptability study in Burkina Faso and Cote d'Ivoire. We found that the WHO SCC was feasible and acceptable for healthcare providers but the tool need to be modify (15, 17).

The implementation of the mSCC involved introducing the mSCC at four regional hospitals in each country. The mSCC was introduced in two formats: A3 (type of standard paper size measuring 297 millimeters in width by 420 millimeters in length) and large display Kakemono (vertically hanging scroll). The A3 format was intended for inclusion in the mother's chart, while the kakemono was to be displayed in the delivery area at a location agreed upon by the staff. The healthcare providers had to use the tool for six months from January to June 2023.

To support the use of the tool, three strategies were employed, which included engaging key stakeholders, conducting cascade training for healthcare providers, and providing peer-coaching support to the trained healthcare providers.

1. Stakeholders engagement: Stakeholders’ engagement occurred before the study's commencement. The research team in each country held meetings with local and central health authorities to introduce the study objectives and procedures briefly. They also discussed the cascade training and coaching-based approaches.
2. Cascade training (a training methodology where information or skills are disseminated through multiple layers of trainers). At each intervention site, the head medical officer of the maternity ward and the most experienced midwife were selected to attend a two-day orientation training on the mSCC and childbirth quality of care. They subsequently trained their respective healthcare providers, on the job site, on using the mSCC during a one-day orientation session to obtain their buy-in.
3. Coaching based approach: The coaches acted as on-site coaches at the facility, responsible for overseeing and following up the daily use of the mSCC.

## 3 Methods

### 3.1 Conceptual framework

We used the CFIR conceptual framework to conduct this study. The overarching aim of the CFIR is to predict or explain barriers and drivers (determinants, independent variables) to implementation effectiveness (the outcome, dependent variable) (18). The CFIR was updated in 2022 based on users feedback (19). Overall, updates to the CFIR include revisions to existing domains and constructs as well as the addition, removal, or relocation of constructs. The updated CFIR includes some forty constructs that address different aspects of intervention implementation (19). Some constructs are based on the COM-B Model for behavior change (20). This conceptual framework can help to produce findings to inform stakeholders on improvements to the intervention and its implementation. Additionally, we used the updated CFIR to facilitate the comparison with other WHO SCC implementation programs and studies.

### 3.2 Study settings

The intervention was implementing in four regional hospitals in each country: Burkina Faso (CHR Gaoua, CHR Dedougou, CHR Pissy, CHR Zianiaré), Guinea (CHR Boké, CHR Conakry, CHR Labé, CHR Kankan), and Cote d'Ivoire (CHR Gagnoa, CHR Dimbokro, CHR Abengourou, CHR Bondoukou). This mSCC was introduced in two formats: A3 and kakemono. The A3 format was intended for inclusion in the mother's chart, while the kakemono was to be displayed in the delivery area at a location agreed upon by the staff. The healthcare providers had to use the tool for six months from January to June 2023.

### 3.3 Study design

To identify barriers and facilitators issues, we conducted a qualitative multiple case design using a triangulation of methods and data source in June 2023. The cases were represented by the regional hospitals where the intervention was implemented: four regional hospitals per country. The methods used were individual interviews.

### 3.4 Participants

Different stakeholders took part in the study depending on their involvement in adapting and/or implementing the mSCC. For each stakeholder category, a purposive sampling was carried out considering qualifications, position, gender, level of education, and professional experience. Representativeness of the key stakeholders and their respective point of view was sought in the sampling. The distribution of the stakeholders was the following in each country: representatives of maternal and child health program (02), managers of regional hospital (04), heads of maternity services (04), healthcare providers (20 i.e., 05 per regional hospital), and coaches (08). A total of 38 individual interviews were expected per country.

### 3.5 Data collection

Regarding the individual interview, the data collection was carried out in June 2023. The interviews were conducted using interview guides containing open-ended, semi-opened, and closed questions. Each guide included themes derived from evaluation criteria and adapted for different groups of stakeholders.

In each country, 4 investigators (2 teams) with a bachelor's degree in social sciences and/an experience in conducting qualitative interviews were trained for 2 days on the overall survey procedures and the content of data collection tools. Role-playing exercises were performed to standardize data collection procedures and questions content. All interviews were recorded on a dictaphone with notetaking. In each country, to confirm saturation, three additional interviews were conducted (these three additional interviews were not included in the analysis).

### 3.6 Data analysis

In each country, all interviews were transcribed verbatim into Microsoft Word from audio recordings by the data collectors. The resulting transcripts were anonymized, accessible only to the research team members. All data were organized in NVivo 14 and coded in two phases. In the first phase, authors in charge of data coding (YWME, DD, SAO and KRK) independently read each transcript several times to identify emerging themes relevant to the research question and coded the quotes that represented each theme. When new themes emerged, the researchers returned to the previous transcripts to re-read and recode them, if necessary. The transcript was coded line by line and each idea was given a name or word summarizing the main idea or concept. The process of developing was iterative and involved numerous conversations among the research team. They compared their codes in regular meetings to review and refine their codes, and to discuss emerging themes. Ten interviews were inter-coded before agreement reached and the percent agreement was 80% (number of codes that all coders agreed on divided by the number of total coded sections). The final categorization was approved by all the research team members during a meeting. Any discrepancies were solved in a systematic and transparent manner throughout several discussions and online meetings of the research team.

Regarding the second phase, after the coding process, a workshop was conducted in September 2023 in Burkina Faso with the research teams of the three countries to analyse the qualitative data. Thematic analysis was conducted using both inductive and deductive approaches. The deductive approach was employed using the updated CFIR to structure the results systematically. Each emerging theme was mapped to relevant constructs within the updated CFIR model, considering how each theme related to intervention characteristics, inner and outer settings, individual characteristics, and processes. Triangulation of data sources was performed to enhance the validity of the findings.

## 4 Results

### 4.1 Participants

Thirty-six participants took part in the study in Cote d'Ivoire and thirty-eight in Burkina Faso and Guinea. Most participants were female in the three countries. Participants over 45 years old were particularly numerous in Guinea (47.4%) and Cote d'Ivoire (38.9%) compared to Burkina Faso, indicating probably higher professional experience in these countries compared to Burkina Faso (23,7%). More than 40% of participants in Burkina Faso and RCI have more than 10 years of experience, while this figure reaches nearly two-thirds in Guinea (65.8%), highlighting a more experienced workforce in this country. The detailed participants' characteristics are summarized in Supplementary File S1.

### 4.2 Barriers and drivers

Seventeen themes emerged as drivers and seven as barriers to the mSCC implementation. We mapped the identified drivers and barriers to the CFIR constructs that best captured their nature and influenced the implementation process. All the themes identified are displayed in Supplementary File S2.

#### 4.2.1 Innovation characteristics

This domain assesses the characteristics of the mSCC that may influence its implementation. Five drivers (a reminder for routine work, clarity of the mSCC, simplicity of the mSCC, facility of use, good design combining training and coaching) were identified and embedded in the following constructs: relative advantage, complexity and intervention package and design.

##### 4.2.1.1 Innovation relative advantage

In the three countries, only one theme (a reminder for routine work) emerged as a driver embedded to this construct. All participants indicated that, although the evidence-based practices described in the mSCC are already included in existing protocols and guidelines, they see the tool as a reminder that brings together evidence-based practices and enables them to structure their work. For them, this is a significant advantage for their routine work, as this healthcare provider stated:

“With the checklist, we're better able to visualize all the gestures to be performed on the parturient. It allows us to catch up on things we've forgotten to do when we're in situations where there are a lot of women to deal with."(BF, HP2)

##### 4.2.1.2 Innovation complexity

When asked about the complexity of the tool, all respondents in the three countries reported that the content is clear, simple, easy to understand and apply, as expressed by one of them:

“Most of the items in the pause points are easy to understand, everything is mentioned from the mother’s admission to her discharge, the tool is very simple and easy to use"(RCI, HP 11)

Another healthcare provider pointed out the facility of use:

“For me, it’s simple since it’s all about ticking off the points. I said everything is detailed, I don't see any points that are complicated” (BF, HP32)

##### 4.2.1.3 Innovation design

The mSCC was introduced into two formats (A3 format and Kakemono) associated with training and coaching. The respondents felt that both formats associated with training and coaching influenced positively the implementation of the tool, as stated by this participant in Cote d'Ivoire:

“Whether A3 or Kakemono, both are practical and useful. The A3 format is portable, and the Kakemono offers an excellent visual effect of good practice. So, we had the Kakemono as a visual in the delivery room, and we could go wherever the parturient was with the A3 format”. We were also able to benefit from training and coaching, which was very useful (RCI, HP 15)

#### 4.2.2 Outer setting domain

This domain includes constructs that interact with different levels outside an organization**.** Under this domain, the participants in the three countries identified two facilitators: consistency with existing guidelines and interaction central level/research team/implementation actors

Consistency with existing guidelines is embedded to Policies and Law construct. This construct captures factors such as legislation, regulations, professional group guidelines and recommendations, which could influence the implementation of the mSCC. When asked about policies and laws that could negatively or positively influence the mSCC implementation, all the participants in the three countries agreed that the tool is consistent with the current directives of the Ministry of Health, and this facilitated its buy-in as reported by this coach in Guinea:

“The fact that the checklist is aligned with the directives and that it has been adapted to our context according to national guidelines further facilitated its use by the midwives’’ (GUI, coach 7)

The theme “interaction central level/research team/implementation actors” was identified under the construct partnership and connection as a driver. This construct highlights relational ties, formal arrangements, and processes that connect outer system and inner organizational contexts. This interaction positively influenced the implementation of the mSCC. It is worth noting the establishment by the research team of bi-weekly online meetings and a WhatsApp group with the coaches, the focal point of the Maternal and child Health Program, and the members of the research team. These platforms were intended to discuss difficulties encountered in the field and find solutions.

“The interactions with the research team and central level during these meetings were truly beneficial for the implementation. Having these meetings at regular intervals and presenting our activities in our respective hospitals consistently encouraged us to improve our performance.” (RCI, Coach 7).

#### 4.2.3 Inner setting domain

This domain includes constructs that interact with different levels within the organization. Five barriers (increased workload, lack of incentives, lack of medicines and other needed supplies, lack of equipment, lack of space for admission and lack of human resources) and three drivers (availability of the mSCC in A3 format, on-the-job training and meetings with hierarchy) were identified and embedded to following constructs of this domain: compatibility, incentives system, available resources, access to knowledge and information.

##### 4.2.3.1 Compatibility

In a context where the workload is already high, several participants stated that their workload was further increased by the implementation of the mSCC, as stated by these participants:

“There is a lot of work and a lot of documents to fill in, such as the patient file, the delivery register and the partograph. With all these tools to fill in, it’s not easy to fill in the checklist at the same time. Midwives sometimes complain that the checklist is an additional workload. (BF, Manager regional Hospital 2)

“They say the volume of activity is too much, and I can confirm that. And there aren't many of them - there are only two midwives in the delivery room, along with an orderly and a hygiene officer. Often, they can perform ten caesarean sections and ten vaginal deliveries, and then complete all the paperwork, including the checklist. So sometimes they don't complete the checklist” (RCI, Coach 6)

##### 4.2.3.2 Incentives system

The lack of incentives was identified as a barrier. Several participants, especially in Burkina Faso and Cote d'Ivoire, indicated that the implementation of the mSCC might have been hindered by the absence of incentives. These healthcare providers described the situation as follows:

“We've already got a lot to do and now we've got to fill out the checklist… we’ve got to think about ourselves a little..I mean a little financial motivation” (RCI, HP28)

We need to be motivated; we need a monthly payment of 100,000f for example. (BF, HP17)

##### 4.2.3.3 Available resources

As for the availability of resources, one driver identified in all three countries was the availability of the mSCC. According to those interviewed, the fact that the tool was available without shortage, facilitated implementation, enabling continuous use, as mentioned by this coach:

“We've never run out of checklists, and we've always received the necessary funds in time to make the copies.” (RCI, coach 3)

However, some respondents expressed concern about the lack of human resources, equipment and materials, as well as space required for optimal implementation of the checklist, medicines and other needed supplies.

The availability of medicines and other needed supplies is an essential condition for the effective use of the checklist. In all three countries, the absence or shortage of certain drugs such as antibiotics, oxytocin and magnesium sulfate, is highlighted by stakeholders as a barrier/difficulty to the use of the checklist. Healthcare providers also face significant challenges in terms of the availability of essential materials and equipment, which affects the effectiveness of checklist use in maternity wards. This situation is highlighted in the following quotes:

“The non-availability of drugs such as magnesium sulphate, oxytocin and anti-retroviral does not allow us to properly manage women and implement the recommendations of the Checklist.” (GUI, HP 11)

Another major barrier identified was the lack of equipment and materials such as delivery kits and sterile gloves as mentioned by these respondents:

“At times we are short of delivery kits” (RCI, HP36)

“Hmmm! One difficulty we really encountered in using the checklist is the use of sterile gloves. And yet the checklist says to use them frequently."(BF, HP4)

The lack of human resources in some health facilities was also considered as a limit to the implementation of mSCC. According to some respondents, some health facilities already encounter human resources shortage which can be exacerbated by the implementation of other intervention such is the case with the mSCC, as mentioned in these following quotes:

“The lack of human resources here is detrimental to optimal use of the tool.” (GUI, coach 8)

“The checklist is welcome, but we have a staffing problem so after filling in the registers we are tired.” (RCI, HP2)

“It means that we must recruit staff, because when you alone are going to stay from eight o'clock until six o'clock and give birth thirty times, really from the afternoon on you are upset, you skip steps so the number of staff must be increased.” (BF, HP6).

While many participants recognized the benefits of the labor companion presence recommended in the mSCC, some of them pointed out the lack of space as a barrier that limits its implementation. Midwives explained it as follows:

“The space in the labor ward is too small and inadequate to receive labor companions “(BF, HP1)

“Having a companion in the labor ward was a good experience, however due to lack of space, the labor ward was overcrowded. If we want to apply this recommendation, our labor ward should be adapted” (RCI, HP11)

“It’s hard to get companions here, just look at how small the delivery room is!” (GUI, HP17)

##### 4.2.3.4 Access to knowledge and information

Meetings with the hierarchy and on-the-job training were two drivers that emerged from the interviews in the three countries. The end-users of the mSCC accessed knowledge and information through meetings organized by the hierarchy and on-the-job training led by coaches. During these activities, they could obtain digestible information about the intervention and how to incorporate their tasks into work for successful implementation, as mentioned in the following:

“What I really appreciated were the meetings with management to explain the checklist and why we were implementing it. These meetings help to clarify things for us and provide answers to our concerns.” (RCI, HP 27)

“The on-the-job training was very necessary. It helped us to understand the Checklist and the importance of applying it. (GUI, HP 2)

#### 4.2.4 Individual domain

##### 4.2.4.1 High level leaders

This construct refers to individuals with a high level of authority, including key decision-makers, executive leaders, or directors. Some participants from the countries studied noted that many policymakers were genuinely interested in using evidence to improve the health of the population, and this positive attitude encouraged their commitment to implementing the mSCC. The involvement of political decision-makers right from the start of the process, with their participation in adapting the tool to national guidelines, ensured their ownership of the project. The involvement of those responsible for Maternal and Child Health Program in each country was particularly important, as several participants emphasized:

“We were short of certain medicines and supplies needed to implement the checklist, so we called the checklist focal point at the mother and child health program and within a few days we received the products. The central level was really involved and gave us a lot of support”. (RCI, coach 1)

##### 4.2.4.2 Mid-level leaders

This construct is about individuals with a moderate level of authority, including leaders supervised by a high-level leader who supervises others. The commitment of heads of maternity and managers of the regional hospitals was identified as a facilitator. In most of the regional hospitals, they held regular internal meetings or discussed with the staff to identify problems, challenges and explore solutions inherent in checklist implementation, as mentioned by this coach:

“It should be noted that our managers are very involved. It is often during meetings or staff gatherings that we take the opportunity to discuss the Checklist. Each coach brings the checklists of the midwives they supervise, and we review them, discuss, and make recommendations”. (GUI, coach 1)

##### 4.2.4.3 Implementation facilitators

This construct refers to individuals with subject matter expertise who assist, coach, or support implementation. One theme emerged as a driver: the presence of the coach. For many participants in the three countries, the presence of the coaches was important for the implementation success, as explained by one of them:

“A key element in implementing the Checklist has been the availability of coaches who can enhance performance in a sustainable manner (GUI, manager of Child Health Program 1)

“Coaching enabled us to change our behavior and accept certain checklist recommendations, such as the presence of companion in the delivery room” (BF, HP16)

##### 4.2.4.4 Capability

In all three countries, healthcare providers felt confident in their capacity to use the mSCC given the fact that there are no new practices to perform.

“Everything in the tool is reflected in the guidelines. It was adapted to our context and there are no new practices outside what we're used to doing. I felt confident using the tool”. (RCI, HP37)

“I decided to use the checklist because I've noticed that what we're asked to do doesn't deviate from our national guidelines. I was capable to use the tool as there are no new practices to perform” (BF, HP12)

#### 4.2.5 Implementation process domain

The themes under this domain were embedded to the following constructs: “engaging” and adapting.

##### 4.2.5.1 Engaging

Two themes were identified as drivers to the mSCC implementation. These themes were: participation in training and the designation as coach. For the participants these two aspects increased their sense of belonging to the organization and consequently their commitment.

All healthcare providers in the three countries emphasized that the training they attended was essential for enhancing their understanding and skills in EBPs and the utilization of the mSCC. One healthcare provider stated:

“We took part in engaging training that equipped us with the necessary knowledge and skills for implementing the mSCC. It also served as a valuable refresher, allowing us to revisit fundamental aspects of obstetrical care.” (GUI, HP19)

As for the coaches, their involvement as coaches had a positive impact on implementation, as it strengthened their commitment, as stated by this coach in Côte d’Ivoire

“We feel really involved with the coaching, it’s a great responsibility and a certain amount of self-confidence shown by the hierarchy, which boosts us and makes us want the implementation of the checklist to be a success in our center.” (RCI, coach 5)

##### 4.2.5.2 Adapting

This construct refers to the action carried out to modify innovation and/or inner setting for optimal adaptation and integration in the work process. The only one barrier identified under this construct in Cote d'Ivoire was related to the Kakemono format. In context, where there is a lack of space in the maternity ward, the motionless character of the Kakemono often poses a difficulty in terms of positioning in the confined space that can be a barrier to its implementation, so adaptations must be made. One coach explained this difficulty:

“The only adaptation was that of the kakemono, how to place it so that it would be clearly visible to the healthcare providers, and they would feel at ease in the labor room. As the labor room is small, we had to rearrange the layout so that we could place the kakemono properly”. (RCI, Manager Regional Hospital 1)

## 5 Discussion

This study assesses the barriers and drivers to the implementation of a modified version of the WHO SCC, adapted to the national guidelines of three west African countries: Burkina Faso, Guinea and Cote d'Ivoire. The strength of this study lies in the fact that it is the first multisite study carried out in West Africa countries about the WHO SCC and using the updated CFIR as framework. This framework is valuable in systematically identifying barriers and drivers of the mSCC. Data sources triangulation was a powerful tool for enhancing the credibility and validity of findings.

In general, the moderate factors were the same across countries. No specificity emerged from the analysis. The main results showed more drivers than barriers. The main barriers identified by almost all the participants were related in the majority to the inner setting through compatibility, incentives system and available resources.

### 5.1 Drivers to the mSCC implementation

Seventeen themes emerged as drivers to the mSCC implementation. The relative advantage of the mSCC lies in its role as a reminder for routine work. Because evidence-based practices (EBPs) were integrated into existing protocols and guidelines, healthcare providers perceive the mSCC as a valuable tool that is consistent with the existing guidelines, consolidates and organizes these practices. In terms of complexity, the mSCC is considered clear, simple and easy to use. These findings are consistent with those of the formative phase of our project (15) and those of other studies (21–23).

The design of the mSCC, with its two formats (A3 format and Kakemono) associated with training and coaching, has positively influenced its implementation. The dual-format approach was a recommendation of the healthcare providers during the formative and adaptation phase of this multicenter study. The portable A3 format and the visually impactful Kakemono address different needs within the delivery room, enhancing the mSCC's utility and visibility.

In addition, this multifaceted design, combined with training and coaching, provides a comprehensive support system that aids in the practical application of the mSCC. The benefits of coaching and on-the-job training on both individual and organizational level are well demonstrated. Coaching and training practices in medical education have been observed to have beneficial outcomes among medical trainees, including more engagement with self-reflection, enhanced workforce performance, more effective acquisition of new clinical skills, and a greater level of positive well-being (24, 25). Lessons learnt from a global collaboration showed that those who had not received any training and those who lacked supervisory support were more uncertain about the use and success of the WHO SCC (5). In our study, participants underscored the importance of their participation in training and the coaching they benefited from. The strategies enhanced their engagement and those of the coaches to implement the mSCC. In India, coaching and mentoring based WHO Safe Childbirth Checklist programs produced increased adherence to some essential birth practices (26–29).

Moreover, in our study, leadership engagement was also critical in encouraging staff to use the Checklist and ensuring supplies. The engagement of the central level, health authorities and regional hospital managers, who acted as high and middle leaders was essential for the implementation success. The engagement of the key stakeholders is one of the most important step when implementing the mSCC (5, 21, 30).

The role played by the research team as a bridging factor between the end users and the decision makers through Zoom meeting and WhatsApp group also facilitated the implementation. Discussions on these digital platforms enabled implementation actors to take corrective action in good time. In some countries where the WHO SCC was implemented, WhatsApp group was also helpful for training follow-up and to address concerns about SCC implementation in real time (21, 31). Indeed, a peer support WhatsApp group was established for those who participated in the WHO SCC training. Trainers were available on the chat for problem-solving, mentorship and ongoing support as the participants rolled out the WHO SCC (31).

### 5.2 Barriers of the mSCC implementation

Seven themes emerged as barriers to the mSCC implementation. Regarding the lack of medicines, the lack of supplies, the lack of human resources, the lack of space and the increased workload, evidence from other countries showed that the above-mentioned barriers were important factors influencing negatively the implementation of the WHO SCC (5, 12, 15, 23, 30, 32–34).

Moreover, the limited space in the labor room does not allow companionship recommended in the mSCC. Other studies on companionship in labor room also identified the lack of space as a key implementation barrier (35, 36).

Staff shortages lead to an overloaded workload that exposes midwives to unnecessary pressure, which could affect negatively the quality and documentation of the care they provide (37). In our study, increased workload hindered the completion of the mSCC as it was also mentioned in the formative phase of the project (15). Workload, particularly in healthcare facilities with high patient volume, has been observed in other countries such as Uganda, Kenya, Cameroon (5, 12, 30–34). In Uganda and Kenya, low rates of completion at the East Africa Preterm Birth Initiative referral hospitals, which had the highest delivery volumes, underscores this critical implementation barrier (34).

As for incentives, it has been well demonstrated that financial and non-financial incentives increase the quality of maternal and newborn care (38). In our study, some participants identified the lack of incentives as a barrier to the use of the mSCC. In Kenya, in response to low uptake in the first 6 weeks after launch and upon urging by the county leadership, healthcare providers were incentivized (USD$0.50) for each checklist completed (34). However, in a study conducted in Mozambique participants expressed that monetary incentives are not necessary to encourage birth attendants to complete the mSCC. Instead, they would appreciate more non-monetary incentives, such as awards, to recognize their work and importance (32).

As for the kakemono format, it needs to be arranged with attention as its impact strongly depends on its location. If it is not in the right place, this can hinder its use. In a context of limited space, before installing the kakemono, it is necessary to test its legibility and to anticipate as far as possible any visual obstacles that may impede the healthcare provider's view.

### 5.3 Implication for policy and practice

#### 5.3.1 Implication for policy

The mSCC was designed based on the national guidelines of the three countries. One of the factors facilitating its implementation is that the tool is acceptable and regarded as a job aid in line with national guidelines. To date, none of the three countries has made use of the mSCC mandatory. To promote the use of the mSCC effectively, it should be framed, for example, by a circular signed and circulated by the Ministry of Health to all health establishments, whatever their level in the health pyramid. Our results on the scalability of the mSCC could be useful and further assist decision-makers in terms of scalability approach and weaknesses to address (manuscript submitted for publication in February 2025).

The findings highlight the importance of adequate allocation of resources to maternal health programs. Policymakers can advocate for increased funding to address shortages in supplies, staff, and infrastructure, human resources and infrastructure shortages (39, 40).

In the design of incentive systems decision makers should align them with local preferences and needs and consider both financial and non-financial incentives to motivate healthcare providers.

#### 5.3.2 Implications for practice

This study yielded interesting findings. Indeed, the results showed that most of the barriers to be overcome are related to the inner context, which will guide programmatic actions. Implementers should incorporate multiple formats of the tool to cater to different needs and environments and ensure that training and coaching are integral parts of the implementation process to support practical use. Health facilities can invest in comprehensive training programs to ensure that staff are well equipped to use the checklist effectively.

Implementers should also engage leaders and stakeholders early in the process to secure their support and ensure resources are allocated effectively. Continuous engagement can also help in troubleshooting and adapting the implementation strategy. It is also important to develop strategies to address shortages in supplies and staff, adapt the placement of tools to fit the available space and consider the physical layout of the implementation environment in the planning stages.

### 5.4 Study limitations

This study provides important insights into the implementation of the mSCC in West Francophone Africa, but some limitations need to be addressed. While data triangulation was used to enhance credibility, the reliance on multiple data sources may still have limitations. For instance, self-reported data from healthcare providers might have been influenced by personal biases or social desirability, affecting the accuracy of the reported barriers and drivers.

## 6 Conclusion

The study on the implementation of a modified version of the WHO SCC, adapted to national guidelines in Burkina Faso, Guinea, and Cote d'Ivoire, provides critical insights into both the barriers and facilitators experienced in these settings. The findings from this research highlight significant programmatic implications that can inform future implementation strategies for similar initiatives in the region. By addressing these barriers and leveraging the identified facilitators, future implementations of similar health interventions can be more effective, leading to improved maternal and newborn health outcomes.

## Data Availability

The raw data supporting the conclusions of this article will be made available by the authors, without undue reservation.
