# Supplementary material for: Exploring barriers and drivers to a modified WHO safe childbirth checklist implementation in three West African countries: a qualitative study using the updated consolidated framework for implementation research
Source: Front Health Serv. 2025 Jun 19;5:1593083. doi: 10.3389/frhs.2025.1593083 (PMC12222196; doi:10.3389/frhs.2025.1593083)
Supplement: Supplementary file 1 [file Datasheet1.docx]

Table 1: Participants characteristics by country

| **Characteristics** | **Burkina Faso (N=38) n(%)** | **Guinea (N=38) n(%)** | **Cote d’Ivoire (N=36) n(%)** |
| --- | --- | --- | --- |
| **Qualification** | | | |
| *Manager of maternal and child programs* | 2(5.3%) | 2(5.3%) | 2(5.6%) |
| *Coach* | 8(21.1%) | 8(21.1%) | 8(22.2%) |
| *Healthcare providers* | 20(52.6%) | 20(52.6%) | 18(50.0%) |
| *Head of maternity* | 4(10.5%) | 4(10.5%) | 4(11.1%) |
| *Manager of Regional Hospital* | 4(10.5%) | 4(10.5%) | 4(11.1%) |
| **Sex** | | | |
| *Female* | 26(68.4%) | 21(55.3%) | 25(69.4%) |
| *Male* | 12(31.6%) | 17(44.7%) | 11(30.6%) |
| **Age group (years)** | | | |
| *25-34* | 10(26.3%) | 0(0.0%) | 0(0.0%) |
| *35-44* | 19(50.0%) | 15(39.5%) | 15(41.7%) |
| *≥ 45* | 9(23.7%) | 23(60.5%) | 21(58.3%) |
| **Experience (years)** | | | |
| < 5 | 9(23.7%) | 5(13.1%) | 5(13.9%) |
| 5-9 | 13(34.2%) | 8(21.1%) | 15(41.7%) |
| ≥10 | 16(42.1%) | 25(65.8%) | 16(44.4%) |
